# Covid-19 demography in France and South Africa: A comparative study of morbidity and mortality in 2020–2022

**DOI:** 10.1371/journal.pone.0294870

**Published:** 2024-02-05

**Authors:** Michel Garenne, Nancy Stiegler

**Affiliations:** 1 Department of Statistics and Population Studies, University of the Western Cape, South Africa; 2 MRC/Wits Rural Public Health and Health Transitions Research Unit, School of Public Health, Faculty of Health Sciences, University of the Witwatersrand, Johannesburg, South Africa; 3 Institut de Recherche pour le Développement (IRD), UMI Résiliences, Bondy, France; 4 Senior Fellow, FERDI, Université d’Auvergne, Clermont-Ferrand, France; Centers for Disease Control and Prevention, UNITED STATES

## Abstract

**Background:**

Covid-19 epidemics raged around the world in years 2020–2022. The dynamics of the epidemics and their mortality varied by country depending on prevention, treatments, vaccination and health status of the population.

**Objective:**

The study compares Covid-19 morbidity and mortality in South-Africa and in France, two countries with similar population size and with reliable reporting systems, in order to better understand the dynamics and impacts of the epidemics and the effects of health policies and programs.

**Data and methods:**

Data on cases, deaths, hospitalizations, vaccinations were drawn from national statistics. Published data on cases and deaths were corrected for undercount.

**Results:**

Results show a different epidemiology in the two countries in the first three years of the epidemic (2020–2022). Incidence was higher in South Africa, and some 44% more people were infected by December 2022 than in France. Mortality and case-fatality were also higher in South Africa despite a favourable age structure. The age pattern of mortality showed higher values in South Africa among the young adults. Young women appeared somewhat disadvantaged in South Africa. Lastly, vaccination appeared to have had no effect on incidence, but a large effect on case-fatality in France.

**Conclusions:**

Despite about the same population size and the same crude death rate at baseline, South Africa exhibited more cases and more deaths from Covid-19 over the 2020–2022 period. Prevention strategies (lockdown and vaccination) appear to have had large impacts on morbidity and mortality.

## Introduction

The Covid-19 epidemic started in China in the late days of year 2019, and spread around the world in the first few months of year 2020 [1]. At global level, the number of cases (incidence) increased rapidly in the first year, peaked in year 2021, and tended to decline in the next year, with less and less cases notified in the second semester [2]. Three years after the onset, it is time to make a first assessment on the dynamics of the epidemic, and on Covid-19 morbidity and mortality over the 2020–2022 period.

The core of the information on the epidemic comes from cases and deaths notified to the World Health Organization (WHO). However this information is of varying quality across the 237 countries and territories included in the WHO database, and a direct comparison country by country would induce much confusion on Covid-19 incidence and mortality [3]. Discrepancies are due primarily to the proportion of cases and deaths notified. These proportions can vary from virtually 0% (some countries do not notify any case nor any death) to close to 100% (small islands with a good health information system). Furthermore, even in the same country the proportion of events notified could vary over time, and was particularly low during in the first months of the epidemic, when biological tests were not yet available or in short supply.

A quick glance at WHO statistics reveals large differences between African and European countries. In proportion of the population, African countries reported 31.8 times less cases and 12.8 times less deaths than European populations. Beyond quality and performances of notification systems, these differences could also be due to a variety of factors. Firstly, several demographic, geographic and economic characteristics of the countries could have an impact: population density, urbanization, trade, internal moves, degree of openness and international travel, economic development (Gross Domestic Product, income per capita), etc. [4, 5]. Secondly, the dynamics of the epidemic itself could vary as a consequence of policies and programs to control the spread of the disease (lockdown, closure of schools, workplaces, restaurants, theatres, etc., and isolation of contagious cases), as well as individual prevention (wearing masks, hand cleansing, etc.) [6–9]. Thirdly, vaccination could also change the patterns of transmission and death: vaccines became available sometimes in 2021 in most countries, but vaccination coverage varied greatly between countries and by age, not counting the fact that various vaccines were utilized with different efficacy and different vaccination schemes. Fourthly, access to care and treatment is an important element of both case notification and death: a proper treatment of cases in a hospital can reduce seriously Covid-19 mortality. In addition, mortality could be affected by the general health status of the population (its underlying level of mortality), as well by co-morbidities (such as obesity, diabetes, hypertension, HIV/AIDS, tuberculosis, etc.) and risk factors (smoking, substance abuse, etc.) [10–12]. Lastly, both incidence and mortality can be affected by the circulating variants of the SARS-CoV-2 virus, some being more transmissible, others being more lethal. In particular the original (so-called wild virus) in 2020, as well as the Delta variant in 2021, appeared somewhat less transmissible and more lethal, while the Omicron variant, dominating in 2022, appeared more transmissible and less lethal [13]. The dynamics of the epidemic in 2022 were also affected by the proportion of the population already infected in the first two years, who were partially immunized against the virus, although cases of repeated infections were frequent. Box 1 summarizes the theoretical framework for analysing morbidity and mortality from Covid-19.

**Box 1. Summary of main factors of morbidity and mortality**

- Population structures: age structure, population density, person to person contacts, travels
- Prevention: lockdown, isolation, hygiene, vaccination
- Risk factors: population health, co-morbidities, other risk factors
- Medical care: acces to care, hospital care, treatment
- Virus characteristics: strain, transmissibility, virulence
- Dynamics of the epidemic: size of the susceptible population

This situation leaves many unanswered questions: What proportion of the population contracted the virus within three years in Africa and in Europe? Was mortality and case-fatality lower or higher in Africa? Did vaccination have an impact on incidence and on mortality? This study aims at shedding some light on this topic and at giving first answers to these questions, by comparing two countries: France and South Africa. These countries have about the same population size, have reliable health information systems, and have published much demographic and epidemiologic data before and after vaccination.

## Data and methods

The numbers of notified Covid-19 cases and deaths come from national agencies, and are reported, in theory daily, to the WHO, as well as vaccinations [3, 14]. In France, case reporting was affected by the screening capacity (availability and use of biological tests), and in particular the number of cases was strongly underestimated during the first six months (January-June 2020). Case reporting is considered close to complete thereafter. However, it should be noted that they could be affected by omissions (people who were not tested or people who used self-testing), as well as by double counts (people could be tested twice for the same episode, or could contract the disease twice or several times). In France, as will be seen below, the number of cases and deaths reported by Santé Publique France (SPF) [15] is not identical to that reported to WHO, but differences are small, and mainly due to cases occurring in institutions for the elderly. The total number of deaths reported to the Civil Registration System was also used, as published by INSEE, the national statistical institute [16] and compiled by INED [17]. All three sources were used in this study and compared.

In South Africa, the number of notified cases is obviously underreported (as will be seen below), and the number of notified deaths is considered much below the real number. A team of researchers from Cape-Town (University of Cape-Town and Medical Research Council) attempted to estimate the number of Covid-19 deaths by studying in great details the variations of mortality during the peaks of cases [18–22]. The method of estimation is complex because not only there are serious differences between deaths attributed to Covid-19 and excess mortality, but also because deaths are often reported with a delay of several weeks or several months (late reporting), and because the discrepancies vary by province. In this study, the number of deaths considered for the final analysis was that estimated by the Cape-Town team, with is approximately three times that officially reported to the WHO.

For estimating the number of cases, seroprevalence surveys were used. These surveys are based on a variety of testing procedures and testing kits, with different characteristics of sensitivity and specificity, and can be based on very different groups (sample of households, sample of health personnel, blood bank, etc.). A web site, SeroTracker, reports sero-surveys conducted around the world, and published formally or informally [23]. In France, sero-surveys are useless, because based on very specific and biased samples (in particular sick persons, health personnel, etc.). In South Africa, several sero-surveys conducted in the general population could be used to estimate the proportion of the population already infected by SARS-CoV-2. The PHIRST cohort is the most useful: this cohort, originally designed for other diseases, was in place when Covid-19 arrived in South Africa, and was able to monitor the spread of the disease since the first months [24–26]. It includes two cohorts of about 600 persons of all ages, one in a rural area (Agincourt), the other in an urban site (Jouberton). They were monitored nine times between July 2020 and November 2021, providing a precise information on the spread of the disease over time. In addition, two sero-surveys were conducted in Gauteng, the most populated province of South Africa, on large representative samples of the population: one in December 2020 (6332 individuals), and a second one about a year later (7010 individuals), just before the arrival of the Omicron variant [26, 27].

For the final estimation of cases and deaths, the following corrections were made. In France, the number of reported cases in the first 6 months (January to June 2020) was multiplied by 10.5, in order to match the number of deaths, keeping constant the case-fatality found in the next 6 months (14.1 per 1000). The number of reported deaths was that published by the SPF. In South Africa, the number of reported cases was multiplied by 13.4, to match the seroprevalence found in mid-November 2021, that is 66.1%. The number of deaths was that calculated by the Cape-Town group, and corresponds to 3.31 times that declared to WHO [19].

In addition, the numbers of Covid-19 hospitalizations were those reported by SPF in France and by NICD (National Institute for Communicable Diseases) in South Africa. Other demographic and public health data (population, urbanization, hospital beds, physicians, etc.) were taken from UN agencies, namely the United Population Division (UNPD) and the World Bank Development Indicators (WDI) [28, 29]. Standard ratios were calculated per population and per case to compare the two countries.

## Results

### Background

The two countries used for the comparison, France and South Africa, had about the same population size (65.1 and 58.6 million), and about the same crude death rate (CDR) 9.4 per 1000 in year 2019, just before the epidemic [28]. They differed in a number of demographic characteristics: life expectancy was higher in France (82.7 vs 64.1 years), adult mortality was lower (36.2 vs 60.3 per 1000), and important for Covid-19 the population age structure was much older in France, with 26.4% of the population aged 60 years and above, vs 8.7% in South Africa. As a result the impact of the age structure on Covid-19 mortality is considerable. Applying the age-specific death rates from Covid-19 in France (April 2020 to April 2022) to the age structure of South Africa implies a 4.23 times lower mortality (237 versus 1001 per million). If population size and crude death rate were similar, the age structure of the south-African population would lead to 4.23 times less Covid-19 deaths, other parameters being equal (Table 1).

**Table 1:** Background information on France and South Africa.

	France	South Africa	Ratio ZA/FR
*Demography*			
Population (2019) (million)	65.130	58.558	0.90
Crude death rate (2019) (per 1000)	9.36	9.40	1.00
Death rate age 60+ (2019) (per 1000)	36.2	60.3	1.66
Proportion population age 60+ (per 100)	26.4%	8.70%	0.33
Age structure effect on Covid-19 death rate (per million)	1001	237	0.24
*Public health*			
Physicians per population (per million)	3267	905	0.28
Hospital beds / Population (per 1000)	5.91	2.30	0.39
*Covid-19*			
Vaccination coverage (Nov 2021) (percent)	77.7%	18.8%	0.24
Periods of lockdown (days) Over 2020–2022 (1096 days)	131	218	

Source: Demography: WPP-2019; Public health: WDI-2019; Other: see text

The two countries also differed in public health infrastructures: France had 3.61 more physicians per capita (3267 versus 905 per million), and 2.57 times more hospital beds (5.91 vs 2.30), so one could expect better access and hospital care in the first case. France reported 131 days of lockdown in three periods, when South Africa reported more days (218 days) and more periods, but comparison here is difficult because strategies were not identical, and probably also because compliance was likely different.

### Epidemic waves

**Fig 1:**
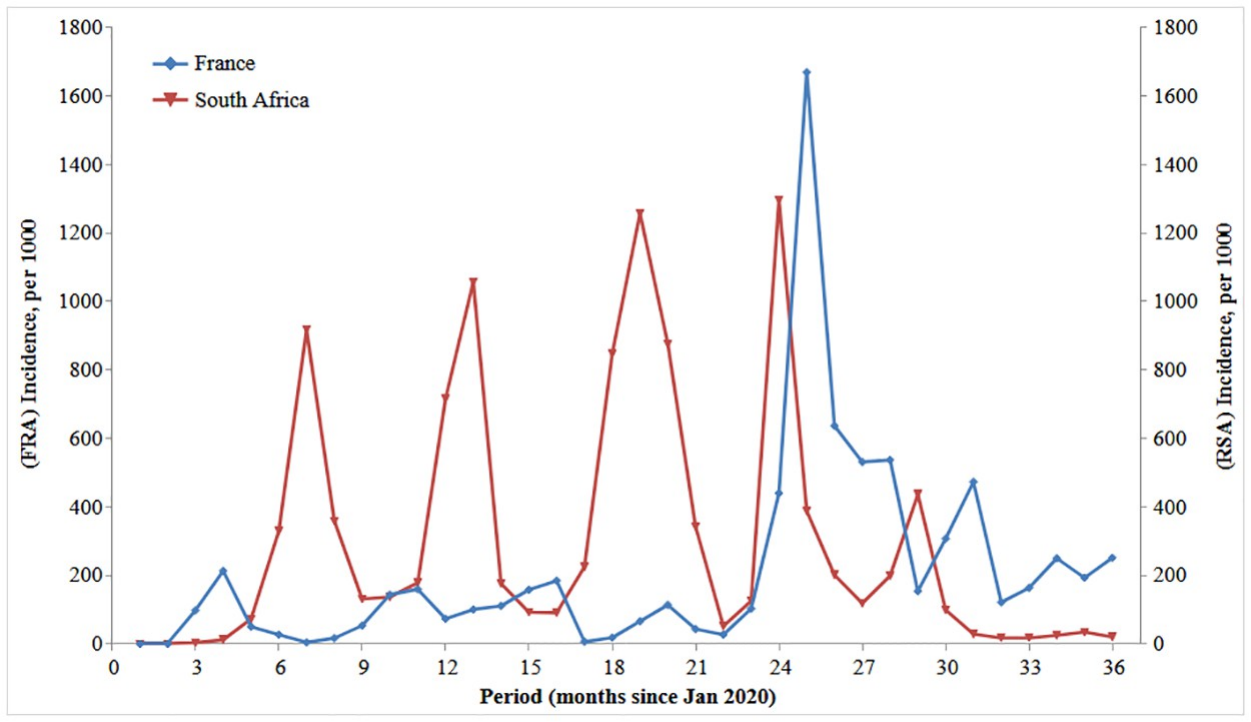
Incidence of Covid-19 in France and South Africa, January 2020 to December 2022.

The epidemiology of the Covid-19 epidemic differed between the two countries. Although SARS-CoV-2 variants were the same and appeared in the same sequence and at about the same time, the number and the size of the epidemic waves differed: France had nine waves, smaller in the first two years, followed by a huge wave in early 2022, whereas South Africa had five waves, the first four being very large, the fifth one in the second semester of 2022 being of smaller size (Fig 1). The difference between the two countries seems to be due primarily to prevention strategies. The smaller waves in France in 2020–2021 resulted from strong restrictive policies (strict lockdown in particular), and the large wave in early 2022 from relaxing most restrictions (despite vaccination, which is discussed later). The South African waves appear closer to a natural course of the epidemic, and closer to what is found in other African countries, as for instance in West-Africa [30]. The smaller wave in the first semester of 2022, and the very low incidence in the second semester (in contrast to France) seems to be due to the saturation of the epidemic, expected because a large proportion of the population has already been infected (estimated about 90% in December 2022).

### Size of the epidemic

France declared 39.3 million cases by December 31, 2022, to which one should add about 2.0 million cases not notified in the first semester of 2020, a total of about 41.3 million cases, which implies that about 62.3% of the population had contracted the virus by the end of the third year. In South Africa, according to seroprevalence surveys, the proportion of the population already infected by mid-November was 66.1%, which implies 90% by December 31, 2022 and some 54.3 million cases. Compared with IHME estimates updated on November 15, 2021, these estimates are somewhat below for France (10.9 vs 15.8 million), and somewhat above for South Africa (43.1 vs 38.6 million) [31–33].

South Africa appears to have had a larger epidemic, probably because France was able to limit the spread of the disease by strong restrictive policies during the first two years, although not in the third year. The few reliable seroprevalence surveys available in France show an infection rate of about 6% in late 2020, roughly matching the numbers obtained from declared cases after correction, whereas seroprevalence surveys available in South Africa indicate a much faster spread of the disease. The best evidence in South Africa comes from the PHIRST cohorts followed by teams from Witwatersrand University in two sites, one rural (Agincourt) and one urban (Jouberton): they show a steady increase in seroprevalence since August 2020, reaching high values of 59.8% (rural) and 69.9% (urban) by November 2021, just after the fourth wave [24–26]. Other population based studies in Gauteng, the most populated province, show the same spread of the virus: 19.1% already infected by December 2020 and 73.1% by November 2021, just after the fourth wave [26, 27]. There is therefore little doubt that the size of the epidemic was about 45% larger in South Africa (approximately 90% infected versus 62% in France).

### Incidence and mortality

Assuming that these estimations of cases and deaths are reliable, comparing basic ratios becomes possible. Incidence (calculated as cases per person-year) was 209 per 1000 in France, against 304 per 1000 in South Africa, 1.46 times more. Mortality was 0.82 per 1000 (deaths per person-year) against 1.90 per 1000 in South Africa (2.32 times more). Case-fatality (deaths per 1000 cases) was 3.93 in France and 6.25 per 1000 in South Africa, (1.59 times more). This result was surprising, since the age structure was so much in favour of South Africa. It seems therefore that age-specific case-fatality (not available) was much higher in South Africa, possibly in a 6 to 1 ratio. This could be due to higher underlying level of mortality, to less access to care, to lower quality of care, and to a variety to co-factors, in particular to concurrent infectious diseases such as HIV/AIDS and tuberculosis.

### Hospitalizations

The number of hospital admissions for Covid-19 was 1.76 times lower in South Africa, which controlling for number of cases indicates a 2.43 ratio of hospitalization per case. This could be due to the age structure, and does not prove a lower access to care. The ratio of notified deaths (most of them coming from hospitals) to hospital admissions appears lower in France (13.8%) than in South Africa (19.2%). This difference could be due to admission of more severe cases in South Africa, and does not necessarily reflect the quality of care. In particular, the proportion of deaths occurring in hospitals is lower in South Africa (30.6%) than in France (82.1%), suggesting better access to care in the latter, and probably better screening for Covid-19 (many Covid-19 cases and deaths seem to remain undiagnosed or at least not notified in South Africa) (see Table 2).

**Table 2:** Comparison of Covid-19 demography in France and South Africa, 2020–2022.

	France	South Africa	Ratio ZA/FR
Nb epidemic waves	9	5	
*Notified events*			
Covid-19 cases	38 226 681	4 048 580	
Covid-19 deaths	158 378	102 568	
*Estimated events*			
Covid-19 cases (million)	~ 41.3	~ 54.3	1.31
Covid-19 deaths	~162 000	~ 343 000	2.09
% infected by 1/1/2023	~62%	~90%	1.44
Incidence / 1000	209	308	1.46
Case-fatality /1000	3.93	6.25	1.59
Death rate / 1000	0.82	1.90	2.33
*Hospital admissions and deaths*			
Admissions	960 945	545 505	0.57
Deaths	132 985	104 541	0.79
Deaths / Admissions	13.8%	19.2%	1.38
*Ratios per population*			
Admissions / Cases	2.4%	1.0%	0.43
% Deaths in hospital	82.1%	30.6%	0.38

NB. Cases and deaths notified to WHO by 01/01/2023. Estimations: see text.

### Effect of vaccination

According to available statistics, vaccine coverage was much higher in France than in South Africa. In France, vaccination started in early 2021, was conducted with different vaccines, with 1, 2 or 3 doses. By October 2021, some 77.7% of the population was considered vaccinated [34]. In contrast, only 18.8% of the South African population was considered vaccinated at about the same time [26]. Even though the comparison is difficult because both vaccines and calendars were different, it is clear that the French population received more vaccines.

The comparison between both countries is pursued by considering the period before October 2021 (21 months pre-vaccination) and after October 2021 (15 months post-vaccination). The impact on incidence was unclear, and difficult to delineate. In France, the epidemic wave occurring just after vaccination was the largest ever recorded, probably because of the large size of the susceptible population (never infected by the virus) and by the relaxing of prevention policies (no lockdown in particular). This situation shows that the vaccines had virtually no effect on transmission (as expected). In South Africa, the fifth wave occurring after vaccination was the smallest, probably because of the smaller size of the susceptible population. In France, incidence post-vaccination was multiplied by 5.0 compared with pre-vaccination, while it was almost halved (RR = 0.54) in South Africa. One clearly observes another phenomenon: the impact of the susceptible population, much larger in France, because of lower incidence before October 2021. Note that in 2022, the same Omicron variant was circulating in both countries.

The effect of vaccination on case-fatality was clearer. In France, case-fatality dropped from 13.0 to 1.40 per 1000 in the post-vaccination period (RR = 0.108), while it dropped only from 7.45 to 3.22 per 1000 in South Africa (RR = 0.43). The ratio of South-Africa to France was 4.0 to 1, which is consistent with the size of the unvaccinated population (ratio of 4.13 to 1). It seems therefore that the vaccination campaigns had a serious impact on mortality, and that this impact was proportionate to the vaccinated population. Of course, a full proof of the effect of vaccine would require proper data by age, sex, vaccination status and co-morbidity (see Table 3).

**Table 3:** Comparison of Covid-19 incidence and mortality by vaccination period in France and South Africa.

	France	South Africa	Ratio ZA/FR
% vaccinated Oct 2021	~78%	~19%	0.24
Incidence, by vaccination period / 1000			
Before Nov 2021	78	377	4.83
After Nov 2021	390	205	0.52
Ratio After/Before	5.00	0.54	
Case fatality, by vaccination period / 1000			
Before Nov 2021	13.00	7.45	0.57
After Nov 2021	1.40	3.22	2.29
Ratio After/Before	0.108	0.432	4.00

NB. Cases and deaths notified to WHO by 01/01/2023, extrapolated.

### Age patterns of mortality

**Fig 2:**
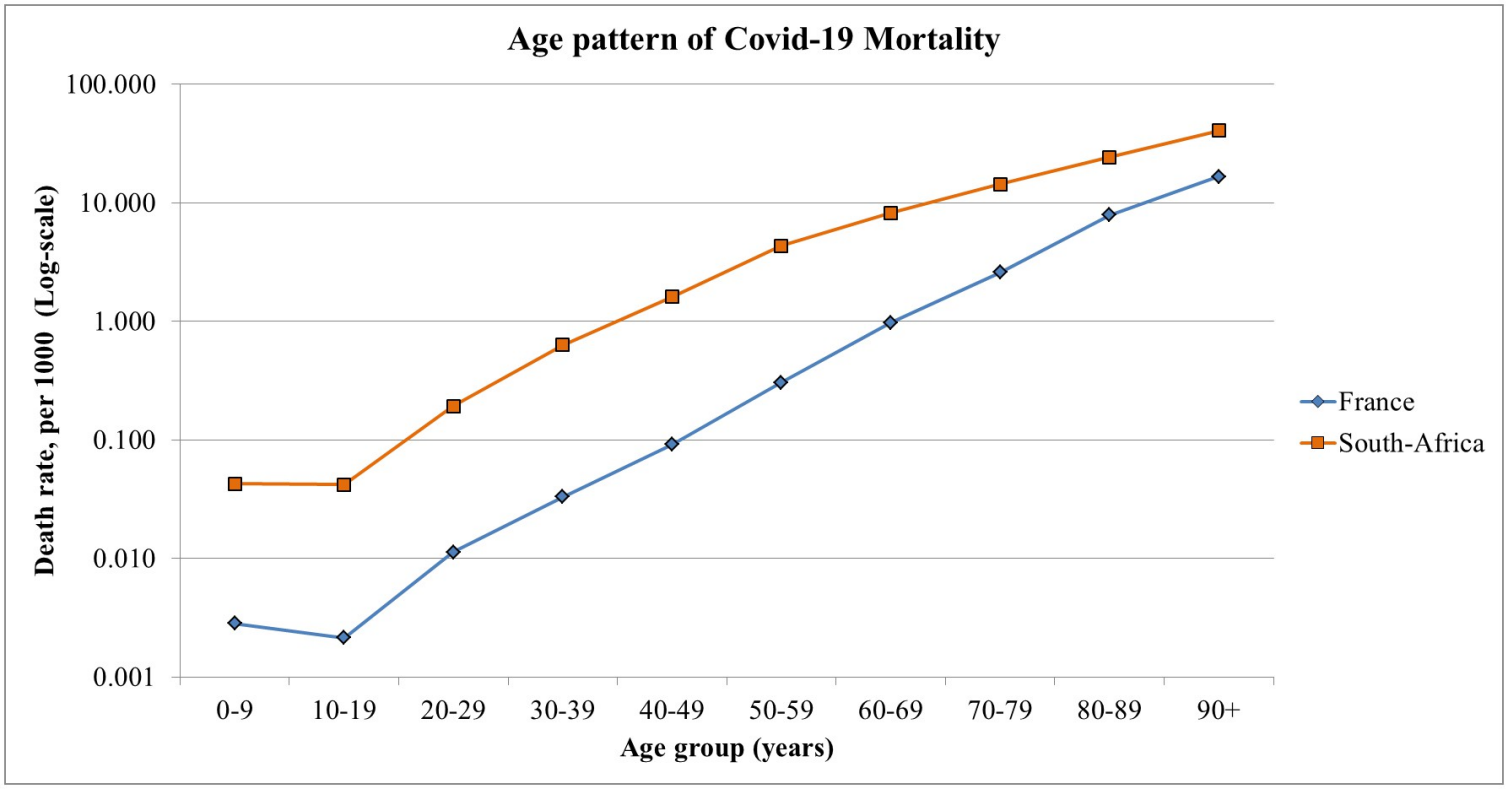
Age pattern of Covid-19 mortality in France and South Africa, 2020–2022.

Age patterns, defined by age-specific death rates from Covid-19 could be compared, as was done earlier in Europe [35, 36]: France, for the 2020–2022 period (updated on April 21, 2022), with South Africa, from hospital based data 2020–2022. Age patterns and other risk factors were also studied in South Africa [37, 38]. Results show age patterns consistent with age patterns of mortality for all cases and for respiratory diseases (Fig 2, Table 4). South African age-specific death rates were much higher for children and young adults, with ratios ranging from 14 to 18 at age 0–59, 6.1 at age 60–79, and 2.6 at age 80+. Due to the large difference in age structures, the ratio was only 1.8 for all ages combined. This excess mortality at young ages is not surprising. Comparing with age-specific death rates for all causes combined during the 2015–2019 baseline period reveals the same differences: ratios of South Africa to France mortality range from 10.2 at age 0–19, 14.2 at age 20–39, 5.6 at age 40–59 and 3.4 at age 60–79 (Table 4). Excess mortality from Covid-19 appears more pronounced than expected from baseline mortality. This could to be explained by the difference in incidence (more cases per population), and possibly reinforced by interactions of Covid-19 with co-factors such as HIV/AIDS and tuberculosis.

**Table 4:** Covid-19 death rates in France and South Africa (per 1000).

Age group	Covid-19 death rates (per 1000)		Ratio ZA/FR	Mortality all causes ZA/FR
	France	South-Africa		
0–19	0.002	0.042	17.2	10.2
20–39	0.023	0.405	18.0	14.2
40–59	0.198	2.721	13.7	5.6
60–79	1.661	10.142	6.1	3.4
80–99	9.806	25.935	2.6	1.1
Total	1.001	1.761	1.8	

Source for all causes mortality = WPP-2019

### Sex differences

In both countries, male mortality from Covid-19 was higher than female mortality, as it is generally the case for respiratory diseases. However, this excess mortality was not verified for all age groups. Female mortality from Covid-19 was somewhat higher in France at age 10–19, and much higher in South-Africa at age 10–39 (as it is the case for tuberculosis and HIV/AIDS). More generally, excess male mortality was more pronounced in France than in South Africa, at all ages except above age 80, with risk ratios of about 1.6 at age 20–79, which could be interpreted as a relative disadvantage of women in South Africa. Comparing again with baseline mortality for all causes, it appears that female mortality from Covid-19 was higher than expected in South Africa at age 15–39 (Table 5). Again, this could be due to interactions with other diseases such as HIV/AIDS and tuberculosis.

**Table 5:** Sex-ratio of Covid-19 death rates in France and South Africa (Male/Female).

Age group	Sex-ratio of Covid-19 mortality		Ratio FR/ZA	Ratio mortality all causes FR/ZA
	France	South-Africa		
0–19	1.15	1.02	1.12 (ns)	0.99
20–39	1.36	0.84	1.62*	1.73
40–59	1.79	1.11	1.61*	1.21
60–79	2.31	1.42	1.63 *	1.34
80–99	1.90	1.90	1.00(ns)	1.04
Total	1.45	0.98	1.47*	

NB: (*) P< 0.05; (ns) not significant. Differences in the sex-ratios were tested by standard T-test on the Logarithm of the ratio.

### Summary impact

**Fig 3:**
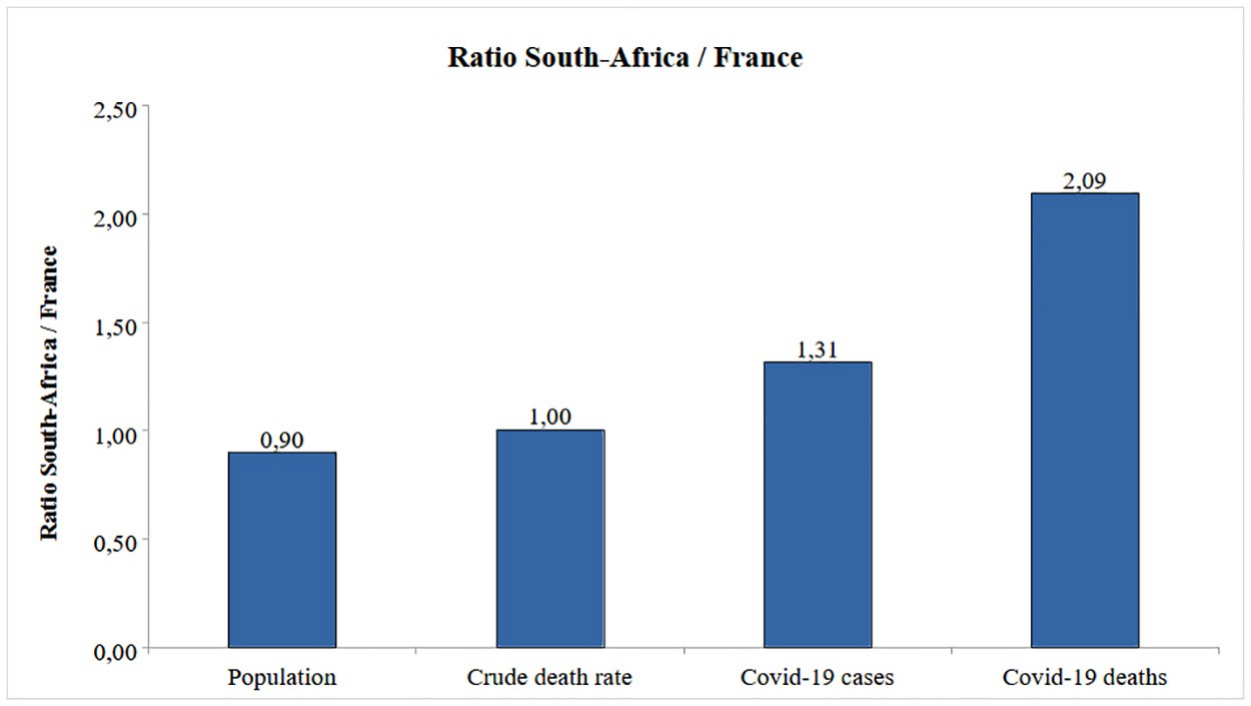
Ratio of basic indicators South Africa / France, 2020–2022.

**Fig 4:**
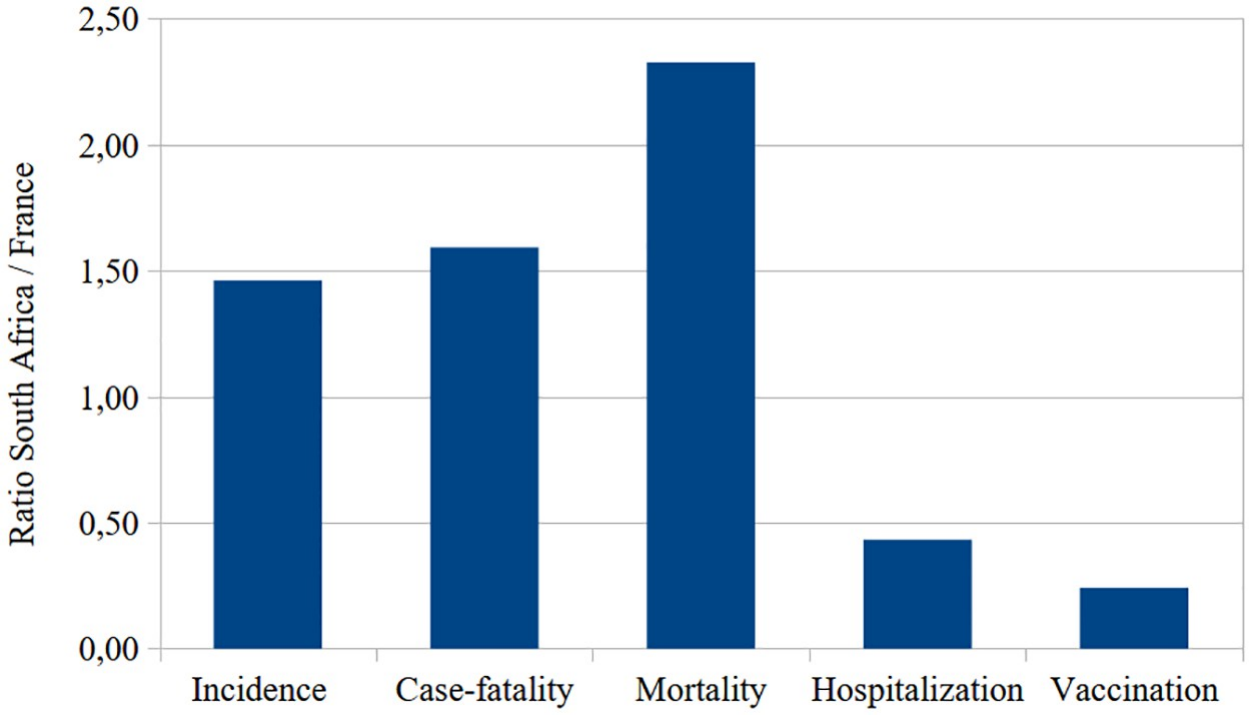
Ratios of selected standardized indicators South Africa / France, 2020–2022.

Figs 3 and 4 summarize the comparison between the two countries. Despite similar population size and death rates at baseline, South-Africa had 31% more cases and 2.09 times more deaths. Lower number of cases in France seems to be due to prevention policies, and higher number of deaths in South Africa to underlying mortality and risk factors and less vaccination, despite a more favorable age structure. In terms of relative indicators (per population), in South Africa incidence was 46% higher, case fatality 59% higher, mortality was 2.33 times higher, whereas hospitalization were 57% lower and vaccination 76% lower. This comparison allowed to better grasp the impact of health policies and programs, and the importance of the underlying health of the population on Covid-19 mortality.

## Discussion

Comparisons are important to better understand the dynamics of epidemics, the factors of mortality, the role of prevention, and the role of vaccination. This study compared the dynamics and population impacts of the Covid-19 epidemics in France and South Africa.

Both countries were severely infected, with a majority of the population infected within three years. Circulating viruses were similar, with similar sequences in the identified variants (original / wild, Beta, Delta and Omicron). In France, the control of the transmission was more efficient, with lower incidence for the first two years, but with a larger outbreak in the third year, still going on in December 2022. In South Africa, the first four waves were more intense and more regular, but incidence was much lower in the third year, and especially in the last six months of 2022. This seems to be due to a more natural course of the epidemic, despite legal actions taken as in France, but with a lower efficacy. It should be noted that strict policies such as lockdown, closing of schools, workplaces, entertainment institutions etc., cannot be sustained in the long run and have be released sooner or later. In the case of Covid-19, strict policies in the first two years, when the virus variants were more virulent, was rather beneficial to France, and the release of constraints in the third year had less impact because the Omicron variant was less lethal, and because vaccines were available.

Overall there were roughly twice as many deaths in South Africa than in France. This was due in part to overall higher incidence (more people infected after three years), to higher proportion of cases during the first two years (when virulence was higher), and to higher vulnerability of the population (measured by higher baseline mortality), and to lower vaccination coverage. These handicaps were in part compensated by a favourable age distribution of the population, with a much lower proportion of older people who concentrate the highest mortality. In terms of treatment, it seems that France had an advantage in the number of people hospitalized, and in outcome after treatment.

The comparison of mortality and case-fatality in the third year gives a clue to the impact of vaccination. Vaccination coverage was high in France when the Omicron variant emerged, while it was still low in South Africa at the same time (October 2021). The drop in case fatality was large in France, while it was lower in South Africa, roughly in the proportion of vaccinated people. Therefore, if vaccines had virtually no impact on transmission, it seems that they had a large impact on case-fatality, and ultimately on the number of deaths. This impact has been already shown in other situations [39].

### Limitations

This analysis was conducted at a broad demographic level, with the aim to provide an order of magnitude of the size of the epidemic and its impacts. Despite much consistency between the two countries, it should be recognized that many estimates have a low level of precision. If the number of deaths can be considered robust, with a good precision in both countries, the number of cases is estimated with hazardous assumptions. In France, one assumed that declared cases were reliable (except in the first six months), which implied that the number of double counts compensated the number of omissions. In South Africa, one assumed that sero-prevalence surveys were reliable (there are serious doubts about the precision of some tests used for measuring antibodies), that results found in local studies could be extrapolated to the whole country, and that the undercount was similar during the three years of the investigation. Another limitation of the study was the lack of precise information on cases by age and sex. One would have liked to be able to compute case-fatality rates by detailed age groups.

Several questions remain open. In particular the role of HIV/AIDS and tuberculosis in South Africa. If these diseases seem to have no effect on incidence [40], they were shown to have an effect on mortality in South Africa [41]. This was suspected in the analysis of mortality levels, age patterns and sex-differences, and the demographic analysis appears consistent with hospital mortality in the Western Cape, with a doubling risk of death among HIV infected people [40].

This study covered only the first three years of the SARS-CoV-2 epidemic. The future remains to be monitored and studied: How will the virus evolve? What will be the responses? Will we be better equipped to treat severe cases? What will be the long-term consequences of persistent infections (long Covid)? What will be the long term consequences on messenger-RNA vaccines? Will the populations adapt to the virus after first selection?

## Conclusions

This study revealed the impact of prevention strategies on morbidity and mortality. By reducing the number of cases during the first two years and by using mass vaccination in the third year, France achieved lower numbers of cases and deaths, despite an unfavorable age structure of the population. This observation could be used in the future for orienting prevention strategies in forthcoming epidemics, as mentionned by other authors [6, 42, 43].
